# Tetrathiomolybdate (TM)-associated copper depletion influences collagen remodeling and immune response in the pre-metastatic niche of breast cancer

**DOI:** 10.1038/s41523-021-00313-w

**Published:** 2021-08-23

**Authors:** Ying L. Liu, Cecilie Liv Bager, Nicholas Willumsen, Divya Ramchandani, Naomi Kornhauser, Lu Ling, Marta Cobham, Eleni Andreopoulou, Tessa Cigler, Anne Moore, Dayle LaPolla, Veronica Fitzpatrick, Maureen Ward, J. David Warren, Claudia Fischbach, Vivek Mittal, Linda T. Vahdat

**Affiliations:** 1 Memorial Sloan Kettering Cancer Center, New York, NY USA; 2 Nordic Bioscience- Proscion, Herlev, Denmark; 3 Nordic Bioscience- Cancer department, Herlev, Denmark; 4 Weill Cornell Medicine, New York, NY USA; 5 Nancy E. and Peter C. Meinig School of Biomedical Engineering, Cornell University, Ithaca, NY USA

**Keywords:** Breast cancer, Prognostic markers, Metastasis, Cancer microenvironment

## Abstract

Tetrathiomolybdate (TM) is a novel, copper-depleting compound associated with promising survival in a phase II study of patients with high-risk and triple-negative breast cancer. We sought to elucidate the mechanism of TM by exploring its effects on collagen processing and immune function in the tumor microenvironment (TME). Using an exploratory cohort, we identified markers of collagen processing (LOXL2, PRO-C3, C6M, and C1M) that differed between those with breast cancer versus controls. We measured these collagen biomarkers in TM-treated patients on the phase II study and detected evidence of decreased collagen cross-linking and increased degradation over formation in those without disease compared to those who experienced disease progression. Preclinical studies revealed decreased collagen deposition, lower levels of myeloid-derived suppressor cells, and higher CD4+ T-cell infiltration in TM-treated mice compared with controls. This study reveals novel mechanisms of TM targeting the TME and immune response with potential applications across cancer types.

## Introduction

Breast cancer is the most common cancer in women, and although women with early stage disease are often treated successfully, metastatic disease is the leading cause of mortality^1^. Efforts to prevent tumor metastases are needed, and growing emphasis is being placed on the role of the pre-metastatic niche and the extracellular matrix (ECM) in tumor metastasis^2,3^.

The ECM refers to the non-cellular component of tissues that dictates cellular polarity and behavior and maintains microarchitecture, structure and function^4^. The ECM consists primarily of various collagens and proteoglycans, which are constantly undergoing turnover and remodeling^4^. In healthy tissue, the degradation of old ECM components and the synthesis, deposition, and cross-linking of new ECM components is maintained in a delicate equilibrium^5^. This equilibrium is often lost in cancer, leading to pathological alterations in the composition and architecture of the ECM that facilitate the hallmarks of cancer metastasis^5^. Emerging data suggest that normalizing ECM composition may be critical in combatting metastatic breast cancer^6,7^.

Copper is an important catalytic cofactor in several biological functions within the ECM and plays a critical role in tumor metastasis^8–10^. Copper is essential for lysyl oxidase (LOX), a key enzyme involved in cross-linking collagen. Collagen is an important component of the ECM^11^. LOX/LOXL2 levels are often increased in cancer, resulting in increased collagen deposition and stiffening of the ECM, thereby facilitating metastatic cell engraftment^12–14^.

Tetrathiomolybdate (TM) is a copper chelator originally used to treat Wilson’s disease^15^, a germline genetic disorder associated with excessive copper accumulation. Pre-clinical studies suggested that TM may also be repurposed to suppress tumor growth and angiogenesis through copper depletion (CD)^16,17^. In a phase I basket trial of TM in metastatic cancers, TM was well tolerated and lead to stabilization but not shrinkage of disease^18^. Additional preclinical and clinical studies have since supported the concept of CD as a therapeutic strategy for cancer treatment^19^.

In a phase II study of women with high-risk breast cancer, treatment with TM after the completion of adjuvant/standard therapies resulted in promising overall survival, particularly for triple-negative breast cancer (TNBC)^20^. Mouse xenograft models of TNBC found that treatment with TM resulted in suppression of lung metastases but no effect on primary tumor^20^. Interestingly, lung tissue from these models had reduced LOX levels as well as reduced collagen cross-linking and fibrillar length, suggesting a role of collagen remodeling in TM-mediated metastases prevention^20^.

Given this, there is a need for novel non-invasive biomarkers of alterations in the tumor microenvironment (TME), particularly examining the ECM and collagen remodeling^21^. Our group has previously shown that serological markers of LOXL2 were significantly higher in patients with cancers as compared to healthy controls^22^. Serum markers of collagen degradation products were also shown to discriminate patients with ovarian and breast cancers vs. healthy controls, suggesting their use as potential biomarkers of disease^23^. Furthermore, Lipton et al.^24^ has shown that said serum collagen degradation products were associated with outcomes in two independent cohorts of patients with metastatic breast cancer. Other studies have also elucidated interactions between collagen framework in the ECM and immune cell infiltration and subsequent immune response^25^, a subject that is becoming more important given the efficacy of immunotherapies in TNBC^26^.

Based on these findings, we sought to examine the impact of CD on collagen and ECM remodeling measured via serum biomarkers in women with high-risk breast cancer treated with TM on a phase II study. We first identified these biomarkers from a panel of collagen markers in an exploratory cohort. We hypothesized that TM-associated CD would inhibit tumor metastases by altering copper-dependent collagen remodeling in the pre-metastatic niche. Using mouse models of TNBC, we then tested the potential for interactions between collagen in the ECM and immune cell infiltration and function.

## Results

### TM study: patient demographics

Between June 13, 2007 and August 1, 2014, 75 patients were enrolled on the trial as previously described^20^. Median age of study entry was 51 (range 29–66) with most being post-menopausal (69%), defined as age > 50 years. The majority of patients were at high-risk of relapse, including 41 (54%) patients with stage III disease and 30 (40%) patients with stage IV no evidence of disease (NED) (Table 1). Of note, 36 (48%) patients had TNBC. The entire cohort received standard chemotherapy either in the adjuvant or metastatic setting prior to enrolling in this study.

**Table 1:** Patient demographics from TM study.

Total patients	*N* = 75
Median age, y (range)	51 (29–66)
Post-menopausal at study entry	52 (69%)
*AJCC stage*	
Stage II, *n* (%)	4 (5)
Stage III, *n* (%)	41 (54)
Stage IV NED, *n* (%)	30 (40.0)
Median tumor size in stage II/III adjuvant pts, cm (range)	2.3 (1.2–7.0)
Median positive lymph nodes in stage II/III adjuvant pts, *n* (range)	6 (1–42)
*Prior disease sites for stage IV pts, n*	
Chest wall	13
Liver	4
Bone only, axilla, lung	3 each
Brain	2
Peritoneum	1
*Molecular subtype, n (%)*	
Luminal A	7 (9.3)
Luminal B	13 (17.3)
Luminal A/B	10 (13.33)
Her2-neu positive	9 (12.0)
Triple-negative	36 (48.0)
*Prior adjuvant antitumor therapy, n (%)*	
Anthracycline and taxane-based therapy	54 (72.0)
Adriamycin-based therapy	6 (8.0)
Cytoxan, Methotrexate, 5-Fluorouracil therapy	5 (6.7)
Trastuzumab	7 (9.3)
Median number of prior chemo regimens in the metastatic setting, *n* (range)	1 (0–3)
*Endocrine therapy on TM, n (%)*	
Tamoxifen	13 (17.3)
Aromatase inhibitors	17 (22.7)
Previous aromatase inhibitors or tamoxifen use	11 (14.7)
Leuprolide	8 (10.7)

TM was well tolerated with few grades 3/4 toxicities including reversible neutropenia 58 events (1.9%), febrile neutropenia 1 event (0.03%), leukopenia 29 events (1.0%), fatigue 6 events (0.2%), and neuropathy 5 events (0.2%) (Supplementary Table 1).

### TM study: survival outcomes

**Fig. 1:**
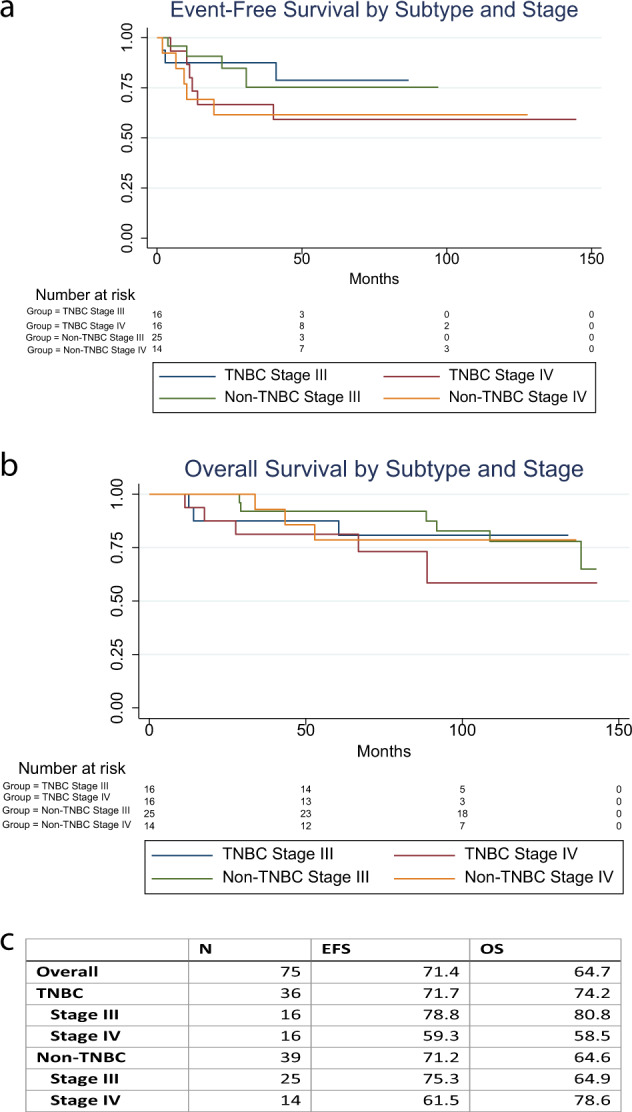
Event-free (EFS) and overall survival (OS) by stage and subtype. Figures depict Kaplan Meier survival curves of event-free (EFS), Panel **A**, and overall survival (OS), Panel **B**, for the entire cohort stratified by subtype (TNBC vs. not TNBC) and Stage (IV vs. IV). Table in Panel **C** depicts numerical values for EFS and OS overall (includes the four Stage II TNBC patients) and by subtype and stage at a median follow-up of 9.4 years.

Of the 75 women enrolled on the study, 74 received at least two doses of TM and were included in the intention-to-treat analysis. Fifty-one patients completed two years of treatment with TM, and 39 women were continued on the TM extension study. At a median follow-up of 9.4 years (3423 cycles of treatment), 14 women experienced disease progression with 17 total deaths observed, of which 14 were related to breast cancer. The EFS was 71.4% and OS was 64.7% for all patients (Fig. 1), and breast cancer specific OS was 79.9%. In those with TNBC, EFS was 71.7%, and OS was 74.2% (Fig. 1). In those with non-TNBC, EFS was 71.2%, and OS was 64.6% (Fig. 1). EFS and OS were lowest in those with Stage IV TNBC (Fig. 1). Among the 4 women with Stage II disease, all had TNBC without disease recurrence and were alive at data cutoff.

### ECM/collagen biomarker exploratory cohort

**Fig. 2:**
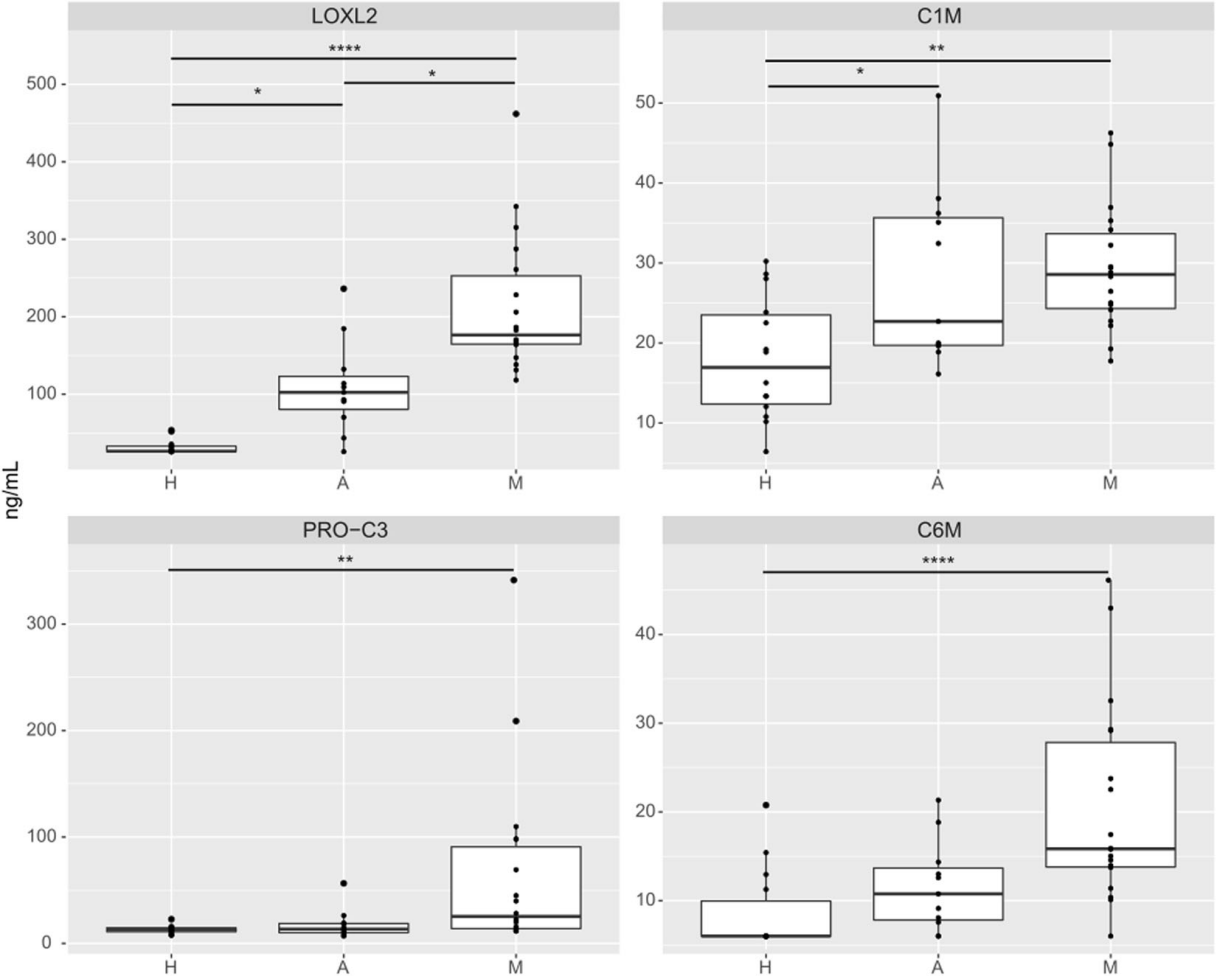
Baseline biomarkers of collagen crosslinking (LOXL2), collagen formation (Pro-C3), and collagen degradation (C1M and C6M) in the exploratory cohort. Tukey boxplots (lines representing 25th, 50th, and 75th percentile with whiskers representing lowest and highest values and *representing outliers defined as 1.5× the 75th or 25th percentile respectively) of LOXL2, C1M, Pro-C3, and C6M levels in those with metastatic (M) and adjuvant (A) disease compared with healthy controls (H). Groups were compared using a Kruskal–Wallis test. Levels of all collagen biomarkers were higher in those with disease, metastatic and adjuvant, as compared with healthy controls. *p*-Values: * < 0.05, ** < 0.01, *** < 0.001, **** < 0.0001.

Baseline serum levels of LOXL2, a marker of collagen cross-linking, were higher in those patients with metastatic disease (*p* < 0.0001) or who were NED and being treated in the adjuvant setting (*p* = 0.04) compared with healthy controls. Compared to those patients being treated in the adjuvant setting, baseline LOXL2 levels were significantly higher than those with metastatic disease (*p* = 0.035), Fig. 2.

Baseline serum levels of PRO-C3, a marker of collagen formation, were significantly higher in those with metastatic disease as compared to healthy controls (*p* = 0.006), but there were no differences between those with metastatic and adjuvant disease, Fig. 2.

Baseline serum C1M levels, a marker of ECM/collagen degradation, were higher in those with metastatic disease (*p* = 0.002) and adjuvant disease (*p* = 0.043) as compared to healthy controls, but there were no significant differences between those with metastatic and adjuvant disease. Baseline serum levels of C6M, a marker of ECM/collagen degradation, were significantly higher in those with metastatic disease as compared to healthy controls (*p* = 0.0002), but there were no significant differences between those with metastatic and adjuvant disease, Fig. 2. Baseline serum levels of DCN-CS, a marker of ECM/collagen degradation, were also higher in those with metastatic disease as compared to those in healthy controls (*p* < 0.001), but there were no significant differences between those with metastatic and adjuvant disease. There were no differences in baseline levels of C4M, VCANM, or NIC (markers of ECM/collagen degradation) between any of the three groups, Supplementary Fig. 1.

Baseline levels of VICM (*p* < 0.01) and CPRM (*p* < 0.01), markers of ECM inflammation, were higher in those with metastatic disease compared with healthy controls. There were no differences in levels between those with metastatic and adjuvant disease, Supplementary Fig. 1.

### Collagen biomarkers in TM patients

Given significant differences between those with active disease and healthy controls in the exploratory cohort, four serum biomarkers (LOXL2, PRO-C3, C6M, and C1M) were further tested in patients on the TM study.

**Fig. 3:**
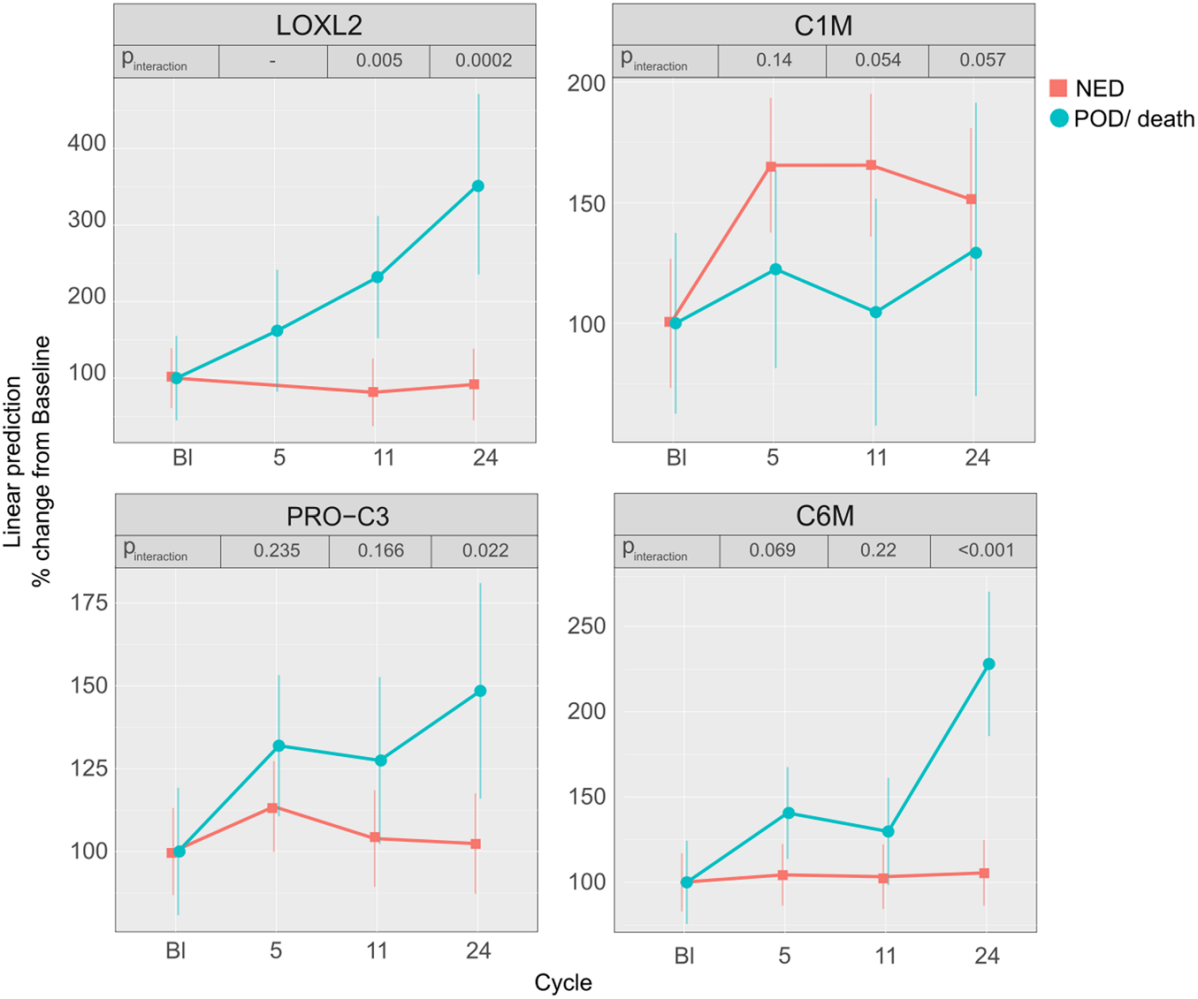
Biomarkers of collagen crosslinking (LOXL2), collagen formation (Pro-C3), and collagen degradation (C1M and C6M) over time in those treated with TM. Figures show LOXL2, C1M, PRO-C3, and C6M levels (estimated marginal means and confidence intervals) from baseline to cycle 24 of TM predicted by the linear mixed effect model in patients with no evidence of disease (NED) and patients with progression of disease (POD) or death. P-interaction (response to treatment × cycle of treatment) is noted for each cycle. LOXL2, PRO-C3, and C6M levels increased in those with POD but not NED while C1M levels increased in those who remained NED as compared to those with POD. There was significant interaction by disease status (NED vs. POD), for LOXL2 levels (interaction *p*-value < 0.01 at cycle 11 and 24) C1M levels (*p* = 0.05 at cycle 11), PRO-C3 levels (*p* = 0.02 at cycle 24), and C6M levels (*p* < 0.001 at cycle 24).

In patients treated with TM, there was a significant interaction between presence of progression/death vs. no disease and changes in collagen biomarkers over time. LOXL2 levels increased over time in those experiencing progression/death; whereas, patients who remained NED had stable LOXL2 levels over time (*p*_interaction_ = 0.005 at cycle 11 and *p*_interaction_ = 0.0002 at cycle 24), Fig. 3. Similarly, PRO-C3 and C6M levels increased over time in those experiencing progression/death but not in those who remained NED (*p*_interaction_ = 0.02 and *p*_interaction_ < 0.0001 at cycle 24, respectively), Fig. 3. Whereas, C1M levels increased in those experiencing NED and remained stable in those experiencing progression/death (*p*_interaction_ = 0.05 for cycle 11), Fig. 3.

**Fig. 4:**
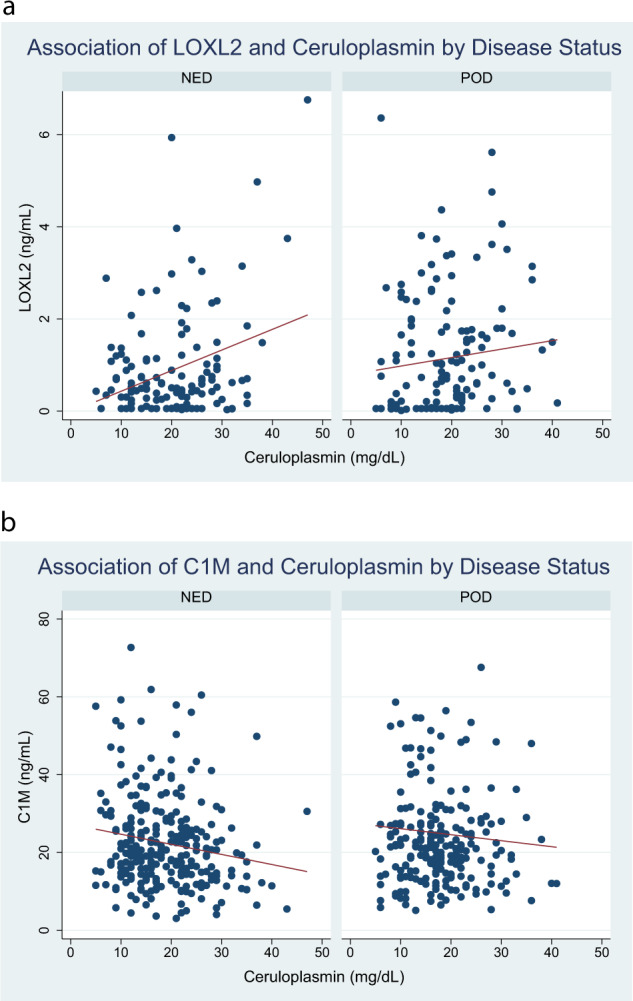
Association of collagen biomarkers (LOXL2 and C1M) with Ceruloplasmin (Cp) Levels by Disease Status. Serum LOXL2 (Panel **A**) and C1M levels (Panel **B**) are plotted against corresponding serum ceruloplasmin level at the same time point for each patient with a line of fit (red). Decreasing ceruloplasmin levels were associated with decreasing LOXL2 (Pearson correlation *p* < 0.001) and increasing C1M levels (Pearson correlation *p* = 0.007) in those who remained without disease (NED), but no significant association was seen in those with disease progression (POD).

We then assessed associations between serum ceruloplasmin levels, a marker of TM-related CD, and collagen biomarkers. As ceruloplasmin levels decreased, LOXL2 levels decreased (Pearson *p* < 0.001) and C1M levels increased (Pearson *p* = 0.007) in those who remained NED only. In those who experienced POD, there were no significant associations between collagen biomarkers and ceruloplasmin levels, Fig. 4.

**Fig. 5:**
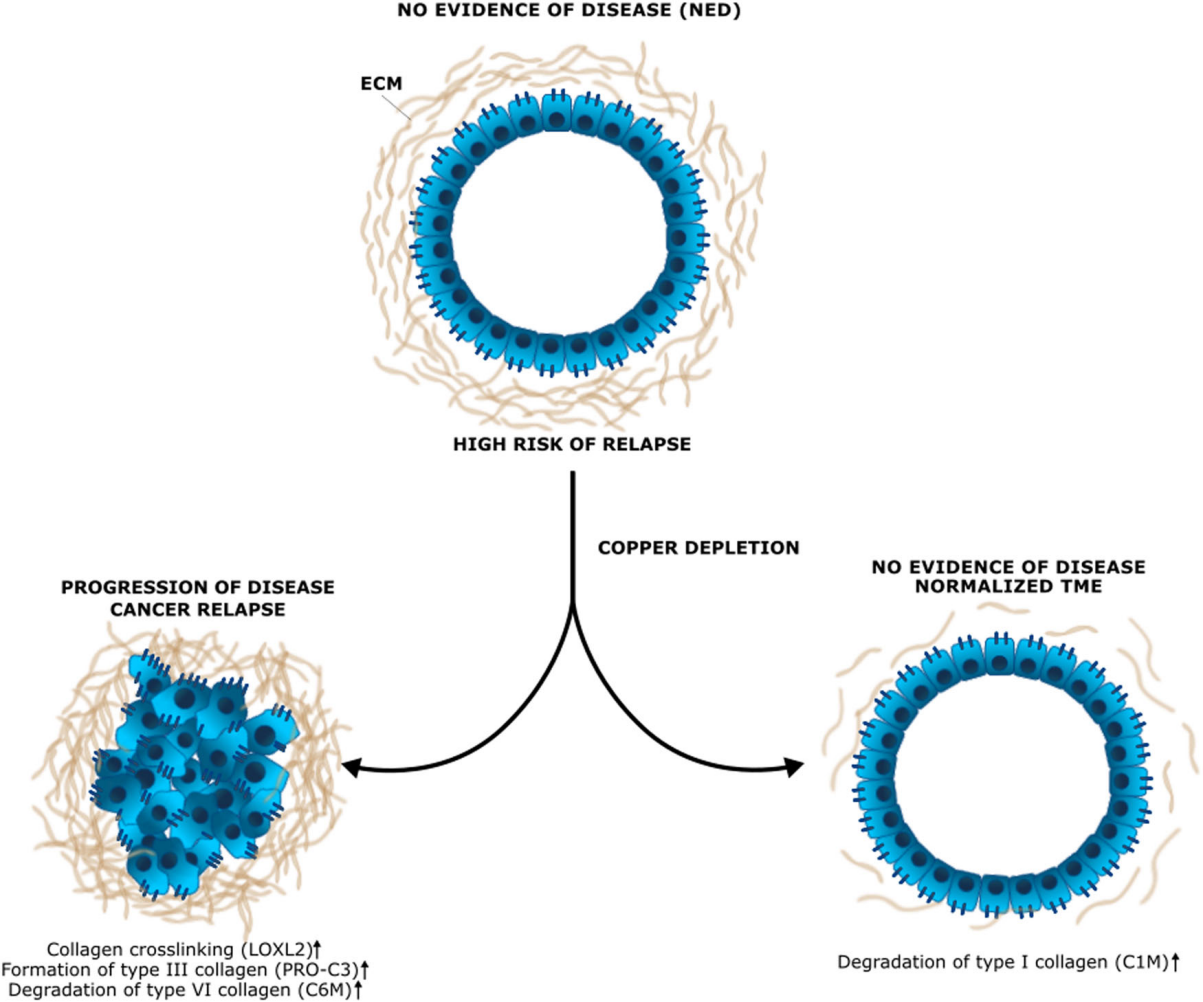
Proposed mechanism of copper depletion’s effects on collagen remodeling in the pre-metastatic niche. In those without disease progression/death, TM-associated copper depletion may lead to decreased collagen cross-linking, as measured by LOXL2 levels, and increased collagen degradation over formation (C1M/Pro-C3), thereby “normalizing” the pre-metastatic niche ECM and preventing tumor metastasis.

This suggests that in patients treated with TM who remained NED, CD may result in decreased collagen cross-linking, as measured by decreased LOXL2 levels, and increased degradation of type I collagen over other types of collagen, thereby “normalizing” the pre-metastatic niche. In those with progression or death, LOXL2, PRO-C3, and C6M, remained high, suggesting ongoing collagen cross-linking and degradation of type III and VI collagen over type I, Fig. 5.

Baseline serum LOXL2 levels were not significantly correlated with C1M, PRO-C3, or C6M levels, Supplementary Fig. 2. At baseline, C6M levels were significantly lower in those who died or experienced progression as compared to those who remained NED (*p* = 0.02). There were no significant differences in baseline levels of C1M and PRO-C3 amongst those who had POD or died compared with those who remained NED, Supplementary Fig. 3.

### Preclinical mouse TNBC models treated with TM: collagen deposition and immune response

**Fig. 6:**
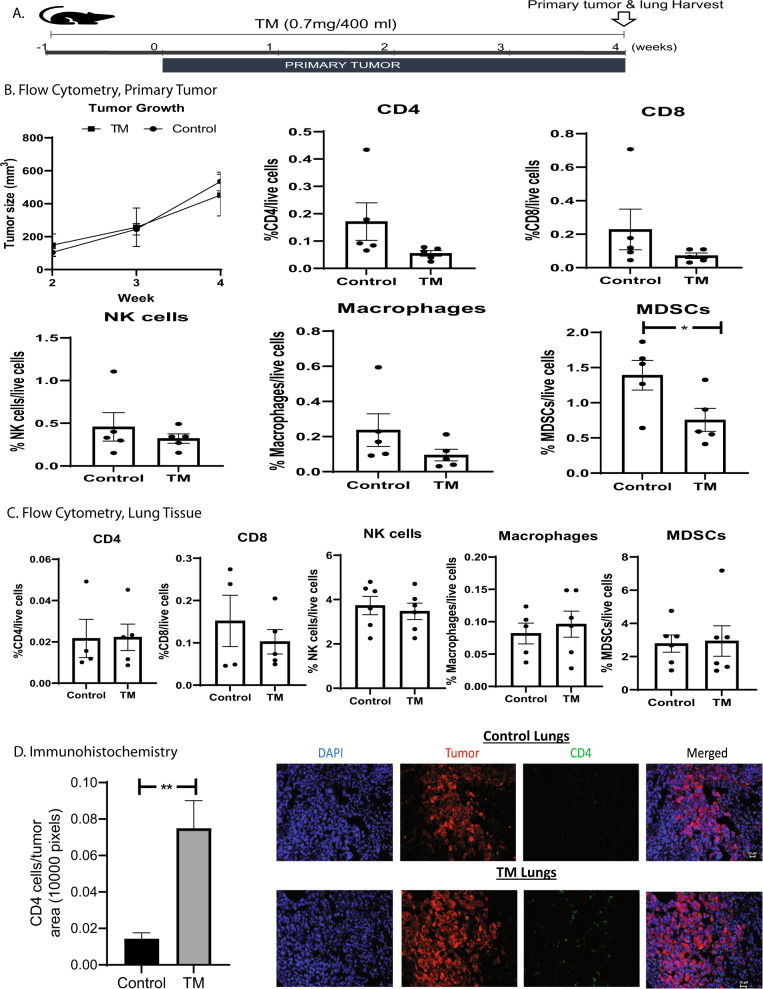
Immune correlatives in orthotopic mouse models of TNBC treated with TM. Panel (**A**) depicts the schematic for an orthotopic, lung metastatic mouse model of TNBC, treated with TM vs. controls. Panels (**B** and **C**) depict immune and myeloid cell populations from flow cytometry in primary tumor (**B**) and lung tissue (**C**), respectively. TM-treated mice had significantly reduced myeloid derived suppressor cells (MDSCs) in primary tumors, as compared to controls. There was a trend towards more NK cells in both tumors and lung tissue of mice treated with TM versus controls. There were no differences in other immune cell populations in the primary tumors or lungs of mice treated with TM vs. Controls. Panel (**D**) depicts results of immunohistochemistry staining and found that tumor infiltration by CD4+ T cells was significantly higher in TM-treated lungs compared with lungs in the control cohort. *p*-Values: *p < 0.05, **p < 0.01. All error bars represent standard error of mean (SEM), and all experiments were run in duplicate.

Given studies showing associations between collagen formation in the ECM and immune response^25^, we explored the effects of TM on immune cell infiltration and signaling and collagen deposition in preclinical models. In orthotopic mouse models of TNBC, 8-week-old C57Bl/6j mice (*n* = 5 for each cohort) were treated with TM in one cohort for a week before tumor implantation in the fourth mammary fat pad (Fig. 6A).

TM-treated mice had fewer myeloid-derived suppressor cells (MDSCs) in primary tumors as compared with controls (*p* < 0.05), although there were no differences in lung tissue. There were no significant differences in other immune cell populations in primary tumor or lung tissue of TM-treated vs. control mice. However, there was a trend towards more NK cells in both the primary tumor and lung tissue of TM-treated mice (Fig. 6B, C).

Immunofluorescence analysis found higher levels of CD4+ T-cell infiltration in the lung tumors of mice treated with TM compared to controls (Fig. 6D). When analyzing effector cytokines secreted by CD8+ and CD4+ T-cells, there was a trend towards higher IFN-g secretion from T-cells in mice treated with TM compared with controls (Supplementary Fig. 4). There was also a trend toward higher PD-1 expression on CD8+ T-cells in TM-treated mice versus controls (Supplementary Fig. 4)^27^.

**Fig. 7:**
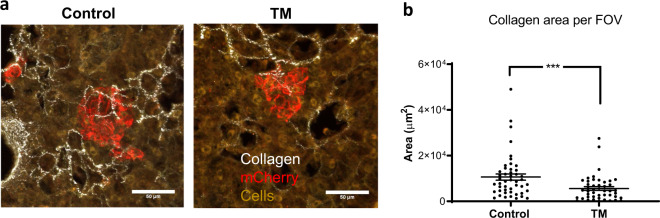
Collagen deposition in mice treated with TM. Collagen deposition in lungs, measured as area per field of view (FOV) on imaging (Panel A), was significantly lower in TM-treated versus control mice, *p* = 0.0027 (*t* test), Panel (**B**). The dots represent individual images, and the bars represent mean and standard error of mean (SEM). Experiments were performed in five control and six TM-treated samples with at least 9 images per sample.

Using a human xenograft model, we compared collagen deposition in the lungs of TM-treated versus control mice. Analysis of collagen deposition by second harmonic generation imaging revealed that lungs of TM-treated mice contained significantly less collagen compared with control mice (*p* = 0.0027), Fig. 7. There was no significant association of enhanced collagen signal around tumor regions (data not shown).

## Discussion

CD with TM was well tolerated and resulted in encouraging EFS and OS in a group of high-risk breast cancer patients. We observed benefit across molecular subtypes (TNBC vs. non-TNBC) and stage (III vs. IV), and we noted prolonged EFS in the majority of patient who were stage 4 NED. This was particularly notable in patients with Stage 4 NED TNBC who had an EFS of 59.3% at a median of almost 10 years of follow-up. This compares favorably with historical controls, as patients with TNBC and Stage 4 disease have a 5-year OS of 11.5% according to SEER data^28^. Because these results are especially notable in patients with TNBC, this pilot trial forms the foundation of a larger randomized adjuvant study that is in development for high-risk TNBC patients.

While potentially promising as a therapy in TNBC, the exact mechanisms of CD as a therapeutic strategy are unclear. Based on pre-clinical data generated in our lab, we further evaluated the effect of CD on collagen processing within the ECM. Our group had previously found that the lungs of TM-treated mice had decreased collagen cross-linking and fibrillar length, resulting in less pulmonary metastasis^20^. To further elucidate the mechanisms of TM on tumor metastasis in patients, a panel of ten collagen/ECM biomarkers were tested in an exploratory cohort, and four (LOXL2, C1M, PRO-C3, and C6M) were identified as being elevated at baseline in those with disease compared with normal controls. These serum biomarkers were then measured over time in patients treated with TM on the phase II study, and we found that LOXL2, PRO-C3, and C6M levels decreased while C1M levels increased in those who remained NED as compared to those who had disease progression. In those who remained NED, LOXL2, and C1M were also significantly associated with ceruloplasmin, a marker of CD related to TM, suggesting a potential connection between TM-dependent CD and collagen processing in those without metastasis. Although this single arm phase II study is limited in its ability to determine causation, analysis of four extreme responders (TM02, TM18, TM26, and TM40) with TNBC who started TM in 2010 or earlier who were NED at the time of this study, supported this hypothesis. During TM treatment, we observed that as ceruloplasmin levels decreased and remained low, C1M levels increased and LOXL2 levels decreased from baseline to cycle 48–60.

Previous studies have linked LOX-mediated collagen cross-linking and a stiffer ECM, which may promote the development of the pre-metastatic niche and tumor metastasis. Cox et al.^14^ found that LOX-dependent collagen cross-linking was involved in creating a growth-permissive fibrotic microenvironment capable of supporting metastatic growth. Other groups have also shown that enhancing interstitial ECM stiffness, potentially through obesity related mechanisms, may also increase malignant potential^29^. In addition, formation, crosslinking and degradation of collagen is increased in patients with cancer and disease progression^22,30^. Furthermore, cancer is associated with a strong fibrotic response with a high degree of collagen formation and cross-linking as well as degradation, which allows migration and invasion of cancer cells through the physical barrier of collagen fibers that compose the interstitial matrix and basement membrane^31,32^. This delicate interplay of collagen formation/cross-linking with degradation may facilitate an important component of cancer invasion and angiogenesis in the pre-metastatic niche.

We hypothesize that TM-dependent CD may facilitate decreased cross-linking, as suggested by lower LOX levels, and increased type I collagen degradation over formation, as suggested by increasing C1M levels and decreasing PRO-C3/C6M levels in those without disease progression. These biomarkers are associated with ceruloplasmin level, suggesting these findings may be due to TM-related CD. We posit that this may alter the pre-metastatic niche in such a way that “normalizes” it and prevents tumor metastasis by creating an inhospitable local microenvironment to support metastatic tumor formation. While collagen traditionally has been viewed to be a passive scaffold for tumor cells, there is growing evidence that collagen may produce biomechanical signals that aid in tumor metastasis^33^. Type I collagen, the most abundant subtype present in human tissue, in particular, has been implicated in metastasis promotion in breast cancer and ovarian cancer and may represent a therapeutic target^34,35^. In addition, the interplay between different types of collagen and ECM fibrosis is extremely complex and poorly understood, and various types of collagen may differentially affect cell signaling and proliferation^36^.

There is also growing emphasis on the importance of immune infiltration and the role of the ECM in facilitating that process. Salmon et al. found that the density of the stromal ECM played a key role in controlling migration of T-cells. In the stroma of human lung cancer tumors, they observed active T-cell mobility in loose fibronectin and collagen regions. Whereas, T-cells migrated poorly in dense matrix areas and were restricted by these fibers. They found that the addition of collagenase increased the ability of T-cells to contact cancer cells, suggesting the importance of the collagen matrix in determining density of the ECM and ability of T-cells to infiltrate tumor cells^25^. Others have found that increased collagen linearization and ECM stiffness and correlated with aggressiveness of breast cancer subtype and was related to infiltration of tissue macrophages and TGF-beta signaling^37^. Although these findings need to be verified in prospective, randomized trials, our study found less collagen deposition in the lungs of mice treated with TM compared with controls. This suggests an additional mechanism in which decreased cross-linking and increased degradation of collagen may create a looser matrix, facilitating immune detection and control of tumor cells.

Our immune correlatives in preclinical TNBC models revealed fewer MDSCs in primary tumors and a trend towards higher levels of effector cytokines in mice treated with TM compared with controls. Presence of MDSCs in TNBC is a negative prognostic marker, and these MDSCs have been shown to activate fibroblasts and recruit monocytes, which alters the TME and increases collagen deposition, potentially facilitating tumor metastases^38^. We also found that treatment with TM significantly increased CD4+ T-cell infiltration into the lungs of mice, a pre-metastatic niche. TM treatment may augment the immune response through decreased infiltration of MDSCs into tumors, potentially leading to less immunosuppression and a more robust response from cytotoxic T-cells, and more infiltration of CD4+ cells into premetastatic sites such as the lungs.

We hypothesize that the decreased collagen cross-linking and increased degradation caused by TM may alter the TME in a way to reduce MDSC infiltration and activation and increase T-cell infiltration into pre-metastatic sites, thereby decreasing metastatic potential. Previous studies have also shown that copper lowering drugs may alter PD-L1 expression and increase CD4+ and CD8+ lymphocyte and NK cell infiltration and activation in neuroblastoma and glioblastoma tumor tissue^39^. Treatment with TM may lead to an immunophenotype favoring activation over suppression of cytotoxic T-cells, potentially facilitating tumor detection and preventing metastases. This is hypothesis-generating as a potential new mechanism for TM’s efficacy in TNBC via immune modulation and should be explored further in randomized clinical trials to verify its efficacy and further explore its mechanism of action. As immunotherapies are gaining traction in TNBC, this finding could also facilitate novel combinations, such as TM with checkpoint inhibitors to improve efficacy in a broader breast cancer population.

Our study uniquely examined the effect of CD on the collagen microenvironment in high risk breast cancer patients and its effect on immune infiltration in a xenograft model of breast cancer. Herein, we propose a novel target for preventing metastases in high-risk breast cancer and explore one of its potential mechanisms involving collagen processing in the TME. We also investigated interactions between immune response and the ECM with regards to copper metabolism and collagen processing in mouse models of TNBC.

The major limitation of our study is the single arm, non-randomized design, which limits our ability to make definitive conclusions about TM’s effects on copper, collagen processing, and immune response. Randomized, placebo-controlled studies are necessary to verify the efficacy of TM in high-risk breast cancer patients and confirm translational findings. Other limitations include testing of multiple endpoints, potentially leading to statistical bias. This is ameliorated in part as we performed adjustments for multiple testing in our exploratory cohort and only examined relevant serum biomarkers in our TM cohort. In addition, although our healthy controls were derived from post-menopausal women, the women on the TM study were primarily post-menopausal, and prior studies have shown that biomarker levels do not differ by age for women before and after menopause^40^. Our study solely focused on collagen processing as one aspect of the ECM affected by CD and is limited in its assessment of the interaction of different types of collagen. In addition, our immune correlates are also limited to mouse models, and more extensive analysis of immune function in patients will be evaluated in future trials. Future studies should explore TM in larger populations, particularly in those with high-risk TNBC, and focus on further elucidating TM’s effects on the ECM, collagen processing and the immune response.

In conclusion, TM is well tolerated and results in promising survival in those with high-risk breast cancer. Part of TM’s mechanism may act through copper-dependent collagen processing, which results in less cross-linking and more degradation, potentially “normalizing” the pre-metastatic niche, increasing immune activation and preventing tumor metastasis. This will be investigated further in a larger cohort of patients with additional emphasis on immune interactions in a randomized phase II clinical trial for high-risk patients with TNBC, which is under development.

## Methods

### Phase II study of TM (NCT00195091)

Clinical grade TM (produced under Good Manufacturing Practice conditions) was purchased in bulk from Sigma Aldrich Chemical Company under IND #71,380 held by L. Vahdat (Department of Medicine, Weill Cornell Medicine, New York, NY). The details of a phase II study of TM in high-risk breast cancer patients has been previously published^20^. The study was conducted under Weill Cornell Medicine protocol #0309006307 and #0611008853, and under Memorial Sloan Kettering Cancer Center protocol #18-023, and appropriate informed consent was obtained from patients

Briefly, patients were considered eligible for the study if they met the following criteria: at least 18 years of age; histologically confirmed stage II TNBC, stage III, or stage IV NED of all molecular subtypes; no radiographic, biochemical, or physical evidence of active breast cancer; more than six weeks from previous therapy including surgery, radiation, chemotherapy, biologic treatment; Eastern Cooperative Oncology Group (ECOG) performance status 0–1 and adequate organ function. For the purposes of this study, the TNBC and basal-like (ER and/or PR ≦ 10% and HER 2 neu–negative) were grouped together as TNBC given recent guidelines^41^. Protocol eligibility was expanded to include patients with stage II TNBC on March 25, 2009. Concurrent hormonal therapy was permitted. HER2-positive adjuvant patients were all required to have completed one year of standard trastuzumab therapy.

Patients were evaluated at baseline and every four weeks thereafter with physical examination and laboratory studies including complete blood count, complete metabolic panel, tumor markers, and research laboratory studies including measures of ceruloplasmin (Cp) levels, a marker of CD. Patients underwent imaging of investigator’s choice, CT of chest, abdomen, and pelvis or PET/CT every six months and as needed to assess for relapse, using Response Evaluation Criteria in Solid Tumors (RECIST)^42^. The National Cancer Institute Common Toxicity Criteria for Adverse Events (CTCAE) version 3.0 were used for toxicity and adverse event reporting^43^.

### ECM/collagen biomarker exploratory cohort

A panel of 10 ECM/collagen biomarkers were assayed by ELISA by at Nordic Bioscience, Herlev, Denmark according to the manufacturer instructions (Nordic Bioscience A/S, Herlev, Denmark). Biomarkers included measurements of formation of type III collagen (PRO-C3, cat. no. 1700), matrix metalloproteinase (MMP) degraded and citrullinated vimentin (VICM, cat. no. 1800), MMP degraded type I collagen (C1M, cat. no. 1000-01), MMP degraded type IV collagen (C4M, cat. no. 1300-01), LOXL2 (cat. no. R1-00), MMP degraded type VI collagen (C6M, cat. no. 1500-01), MMP degraded CRP (CRPM, cat. no. 7000), MMP degraded versican (VCANM, cat. no. 6000), cathepsin-S degraded nidogen-1 (NIC, cat. no. R136-00) and cathepsin S degraded decorin (DCN-CS, cat. no. R1023-00) (Supplementary Table 2) and were tested in an experimental cohort of women with breast cancer receiving systemic therapy to determine the most efficacious biomarkers in distinguishing stage of cancer compared with healthy controls. The details of each assay has been published previously^44–50^.

In brief, the biomarkers ware assayed as follows: a biotinylated synthetic target-peptide were dissolved in assay and added to 96-well streptavidin plates with 100 µL per well. The plate incubated for 30 min at 20 °C and was subsequently washed five times in wash buffer (20 mM Tris, 50 mM NaCl, pH 7.2). Samples or the peptide calibrator was added in duplicates with 20 μL per well, to the appropriate wells followed by the addition of either 100 μL of a horse radish peroxidase (HRP)-conjugated target specific monoclonal antibody or 100 μL of an unconjugated target specific monoclonal antibody. The plate then incubated for 1 h at 20 °C, or overnight at 4 °C, depending on the assay. The plate was then washed five times in wash-buffer. For the assays using unconjugated target-specific monoclonal antibodies, 100 μL of HRP-conjugated secondary IgG antibody diluted in assay buffer was added and the plate incubated for an additional hour and then washed five times in wash buffer. This was followed by adding 100 μL Tetramethylbenzidine (TMB) was added, and the plates then incubated for 15 min at 20 °C in darkness before adding 100 μL stopping solution (1% H_2_SO_4_) stopping the reaction and allowing for the optical density (OD) of each well to be measured at 450 nm with 650 nm as reference. For all assays, intra- and inter-assay variations are <10% and <15%, respectively.

The selection of the patients in the experimental cohort has been described previously^51^. Briefly, 91 women with breast cancer were prospectively recruited from March 2005 to July 2009 at the initiation of adjuvant systemic therapy after surgery in those with Stage I-III breast cancer and at the initiation of a new systemic therapy for those with metastatic disease. Clinical specimens were obtained at baseline (before initiation of systemic therapy), halfway through systemic therapy, at the end of systemic therapy, every three to six months thereafter, and at progression of disease if applicable. Among the 61 women with Stage I-III breast cancer receiving adjuvant therapy, two experienced relapses of their disease. Among the 30 women with metastatic breast cancer, 16 had disease progression, six had stable disease, and 11 had response to treatment. Follow-up is currently ongoing.

Levels of the ten ECM/collagen biomarkers were measured at baseline in eighteen women with metastatic disease, eleven women with adjuvant disease, and fourteen healthy controls from an internal database (Nordic Bioscience) of post-menopausal women without cancer.

### Collagen biomarker analysis in TM patients

The four collagen biomarkers identified in the experimental cohort (LOXL2, C1M, PRO-C3, and C6M) were then tested in 69 of the 75 patients treated with TM on the phase II study. Six patients did not undergo collagen biomarker analyses due to insufficient serum samples.

C1M, PRO-C3, and C6M levels were measured using Nordic Biosciences assays as described above. LOXL2 levels were measured as previously described^20^. Briefly, LOXL2 serum levels were quantitated using an ELISAkit from US Biologicals following the manufacturer’s protocol. One-hundred microliters of patient serum was added in duplicate to the wells of a microtiter plate coated with a biotin-conjugated antibody specific to LOXL2, along with concentration standards. After 2 h incubation, avidin conjugated to horseradish peroxidase (HRP) was added to each well and incubated. TMB substrate solution was added and wells containing LOXL2 biotin–conjugated antibody and HRP enzyme-conjugated avidin exhibited a color change. The degree of color change was then measured with a spectrophotometric plate reader at 450 nm. Concentrations were calculated on the basis of the standard curve. These markers were measured at baseline and over time (5, 11–12, and 24 cycles of treatment, 1 cycle = 4 weeks) in serum samples.

### Statistical analysis

Descriptive statistics for demographic and clinical variables were tabulated for all patients enrolled in the trial. The intent-to-treat population consisted of patients who had at least two doses of TM. The following outcomes were recorded: toxicity attributable to TM, time to progression of disease, overall survival, and serum biomarkers of interest.

Events were defined as recurrence/progression via imaging or pathology, and event-free survival (EFS) was calculated from start of TM to date of event or last follow-up in those without events. Overall survival (OS) was defined from start of TM to date of death (all-cause) or censor date. Breast cancer specific overall survival was defined as deaths related to breast cancer. Kaplan–Meier survival analysis was used to calculate EFS and OS overall and stratified by tumor subtype (TNBC vs. no-TNBC) and stage (II/III vs. IV).

Summary statistics were used to assess range, mean and median levels of each of the biomarkers over time as a percentage of baseline levels. Differences in baseline median biomarker levels were assessed between those with metastatic disease, adjuvant disease and healthy controls using Kruskal-Wallis rank sum test for multiple comparisons of independent samples. The Bonferroni method was used to adjust for multiple comparisons. Linear mixed-effects models were applied to determine the relationship of each biomarker with cycles of treatment and response to therapy. The models included biomarkers (LOXL2, C1M, C6M, and PRO-C3) as outcome variables, cycles of treatment and response to treatment as fixed effects (cycles x response) and patients as random effect. To assess the influence of degree of CD, as measured by serum ceruloplasmin, on the collagen biomarkers, the association of various collagen biomarkers with ceruloplasmin levels was plotted and analyzed using Pearson correlation coefficients, stratified by response to treatment. The linear prediction from the mixed effects models were plotted using the emmeans package (R-software version 3.4.1, package: lme4 version 1.4.2). The statistical analyses were performed using GraphPad/Prism (version 7), STATA (version 16), and R software (3.4.1 version, R Development Core Team, 2017). Two-sided *p*-values < 0.05 was chosen for statistical significance.

### Preclinical studies

All animal work was conducted in accordance with a protocol approved by the Institutional Animal Care and Use Committee at Weill Cornell Medicine. All animal experiments were performed within one week of thawing tumor cell lines. Mouse syngeneic TNBC cell lines EO771 (obtained from Robin Anderson lab, La Trobe University, Australia) and its lung metastatic derivative EO771.ML1 (derived according to standard protocol^52^ at Mittal lab, WCM) were used. Both EO771 and EO771.ML1 have been modified to express mCherry and firefly luciferase. Eight-week-old female C57Bl/6j mice were obtained from the Jackson Laboratory. In an orthotopic tumor prevention model, one cohort of mice (*n* = 5) were maintained on TM for 1 week before tumor implantation. After 1 week, 1 × 10^5^ EO771.ML1 cells in 50 μL HBSS were injected into the fourth mammary fat pad of control and TM-treated group (n = 6 each). Primary tumors were allowed to grow for 4 weeks, then mice were euthanized (by IP injection of ketamine/xylazine, followed by cervical dislocation) and both primary tumors and lungs were harvested for analysis of tumor and, immune populations by FACS. For in vivo T-cell functional analysis, C57Bl/6j mice pre-treated with TM for one week and control cohort were injected with 1 × 10^5^ EO771 cells in 100 μL HBSS by tail vein. Tumors were allowed to grow in lungs for 14 days, mice were then euthanized, and lungs were harvested to assess T-cell function. Only data from mice analyzed for all studies were included in this analysis.

### Primary tumor and lungs flow cytometry staining

Primary tumors and lungs from EO771.ML1 injected mice were digested in digestion buffer (0.05% Collagenase IV, 0.01% Hyaluronidase, 200 units of DNAase 1 in HBSS containing calcium and magnesium) at 37 °C for 30 min. The digestion process was then neutralized with RPMI-1640 media supplemented with 10% FBS, penicillin-streptomycin, and l-glutamine. Single-cell suspensions were obtained as described previously^53^. In short, cells were filtered through 70 μM filters, red blood cells lysed using ACK lysing buffer and finally strained through a 40 μM filter. Cells were stained with fixable live/dead stain, Zombie aqua, for 15 min in PBS on ice. Cells were then washed and resuspended in FACS buffer. For surface stains, samples were blocked with anti-mouse CD16/32 mouse Fc block for 15 min at room temperature, incubated with primary antibodies for 45 min in dark on ice, washed with FACS buffer, acquisition performed on BD Fortessa, and analyzed by FlowJo v10. For T-cell functional analysis, isolated lung samples after digestion were stimulated with PMA (100 ng/mL) and ionomycin (1 μg/mL) for 4 h in complete RPMI at 37 °C in a humidified incubator. Brefeldin A (Biolegend) and Monensin (Biolegend) were added to achieve Golgi blocking. The single-cell suspension was filtered and then stained with Zombie aqua, blocked with anti-mouse CD16/32 and stained for surface markers (CD3, CD4, CD8, and B220) as described. These were then washed with FACS buffer, fixed with Fixation/Permeabilization Buffer (ebioscience) for 30 min in the dark on ice, washed with permeabilization buffer and incubated with primary antibodies for cytokine staining for 30 min on ice. These were then washed three times with permeabilization buffer, resuspended in FACS buffer, and stored at 4 °C for flow cytometric analysis within 24 h. Acquisition was performed using BD Fortessa and analysis using FlowJo v10 (Supplementary Fig. 5).

### Cell surface markers for myeloid and immune population analysis

For distinguishing different myeloid-derived cells, single-cell suspensions from primary tumors were stained with CD45 (clone 30-F11), CD11b (clone M1/70), CD11c (clone N418), F4/80 (clone BM8), Ly-6G (clone 1A8), Ly-6C (clone HK1.4),CD103 (clone 2E7), NK1.1 (clone PK136), CD3 (clone 17A2), CD4 (clone RM4-5), and CD8a (clone 53-6.7). All antibodies were obtained from Biolegend and used at a dilution of 1:100. Macrophages were defined as CD45+, CD11b+, Ly-6C−, Ly-6G−, CD11c−, and F4/80+. Dendritic cells were defined as: CD45+, CD11c+, F4/80−, Ly-6G−, Ly6C−. MDSCs were identified as follows: CD45+, Ly-6G+, CD11c−, F4/80−. NK cells were identified as: CD45+, CD3−, and NK1.1+. CD4 and CD8a cells were respectively defined as (CD45+, CD3+, and CD4+) and (CD45+, CD3+, and CD8a+), **(**Supplementary Fig. 5).

### Immunogenic profile in lungs

For immunogenicity, single-cell suspensions from primary tumors were stained with the following antibodies after stimulation: PD-1 (clone 29F.1A12), TNFα (cloneMP6-XT22), IFNγ (clone XMG1.2), and GzmB (clone GB11). Zombie dye (BV510) was used as a live-dead stain for fixed samples. All antibodies were obtained from Biolegend and used at a dilution of 1:40, except PD-1 which was used at 1:100 dilution.

### Immunostaining and microscopy

For immunofluorescence (IF) staining, 10 μM thick lung sections were air dried in dark at room temperature, followed by blocking and staining with DAPI (1:1000) and anti-CD4 (FITC 1:25, clone RM4-5, Tonbo Biosciences). Fluorescent images for DAPI (nuclei), GFP (CD4), and mCherry (tumor cells) were obtained using a computerized Zeiss fluorescent microscope (Axiovert 200 M), fitted with an apotome and an HRM camera. Images were analyzed using Axiovision 4.6 software (Carl Zeiss Inc.). IF images were quantified using imageJ software.

### Collagen deposition in preclinical mouse model

To study collagen deposition in control vs TM treated mice, we used a human xenograft model in 8-week immunodeficient SCID mice (Charles River). One cohort of mice was treated with TM (*n* = 5). A second cohort of mice (*n* = 5) served as control. Mice were analyzed for Cp activity reduction in the TM cohort, followed by the tail vein injection of 2 × 10^5^ MDA-MD-231.LM2 cells, expressing mCherry and firefly *luciferase*, to resemble an experimental lung metastasis model. Colonization of tumor cells in the lungs was observed by BLI and tumors were allowed to grow for another 2 weeks. The mice were then euthanized as described before. Lung tissues were fixed in 4% paraformaldehyde for 24 h at 4 °C. Paraformaldehyde was then changed to 30% sucrose for another 24 h at 4 °C. These tissues were then then embedded in OCT blocks prior to sectioning. Sections measuring 100 μm were obtained from the lung sections, which were then probed with Rabbit pAb to mCherry (Abcam, Cat#ab167453), followed by secondary staining with fluorescently conjugated goat anti-rabbit antibody (Life technologies, Ref#A11011). mCherry would label all the tumor cells. Multiphoton second harmonic generation (SHG) imaging was used to quantify collagen content in the stained cross-sections of lungs using a custom-built multiphoton microscope at Cornell University’s Biotechnology Resource Center followed by image analysis^29^. A custom-built Zeiss u880 multiphoton microscope was used to visualize collagen fibers by SHG imaging. Images were captured using a W Plan-Apochromat 20×/1.0 Korr DIC M27 75 mm objective. At least nine images per sample were captured, each consisting of a 3D z stack of at least 50 µm in depth. To quantify collagen, images of the 3D stack were projected in the XY‐plane using ImageJ software. Positive area of collagen was quantified within the same field of view after thresholding against a blank background.

### Statistical analysis for in vivo experiments

Analyses of different treatment groups was performed using unpaired *t* tests, with the GraphPad Prism statistical program. *p*-Values < 0.05 were considered statistically significant. Results are expressed as mean ± SE, unless otherwise mentioned.

### Reporting summary

Further information on research design is available in the Nature Research Reporting Summary linked to this article.

## Supplementary information

Supplementary Materials

Reporting Summary

## Data Availability

The data generated and analyzed during this study are described in the following data record^54^. A list of the files containing the data is included on the data record. Authors have chosen not to share the data publicly as they contain ongoing, unpublished work included in other projects. However, the data and full protocol are available upon request from the corresponding author.

## Code availability

The statistical code used during these analyses are available from the corresponding author on reasonable request. STATA (version 16) and R software (3.4.1 version, R Development Core Team, 2017) were used.
