# Supplementary material for: Tetrathiomolybdate (TM)-associated copper depletion influences collagen remodeling and immune response in the pre-metastatic niche of breast cancer
Source: NPJ Breast Cancer. 2021 Aug 23;7:108. doi: 10.1038/s41523-021-00313-w (PMC8382701; doi:10.1038/s41523-021-00313-w)
Supplement: Supplementary file 1 — Supplementary Materials [file 41523_2021_313_MOESM1_ESM.pdf]

Supplementary Files

Supplementary Table 1: TM Adverse Events

**Adverse Event**		**N (%)**	**N (%)**
		**All Grades**	**Grade 3/4**
Hematologic			
	Anemia	324 (10.8)	1 (0.03)
	Neutropenia	418 (14.0)	58 (1.9)
	Febrile Neutropenia	1 (0.03)	1 (0.03)
	Leukopenia	425 (14.2)	29 (1.0)
	Thrombocytopenia	31 (1.0)	0 (0)
Gastrointestinal			
	Sulfur Burps	849 (28.4)	0 (0)
	Nausea	76 (2.5)	0 (0)
	Vomiting	8 (0.3)	0 (0)
	Diarrhea	46 (1.5)	0 (0)
	Constipation	7 (0.2)	0 (0)
	Abdominal Pain	1 (0.03)	0 (0)
General			
	Fatigue	729 (24.4)	6 (0.2)
Neurologic			
	Dizziness	25 (0.8)	0 (0)
	Neuropathy	537 (17.9)	5 (0.2)

Legend: This table depicts adverse events related to TM using CTCAE v. 3.

Supplementary Table 2: Biomarkers of Collagen Remodeling, Nordic Biosciences

#	Biomarker	Specification	Description
**ECM Formation**			
1	PRO-C3	Released N-terminal pro-peptide of type III collagen	Interstitial matrix formation
**ECM Degradation**			
2	C1M	MMP degraded type I collagen	Interstitial matrix degradation
3	C4M	MMP degraded type IV collagen	Basement membrane degradation
4	C6M	MMP degraded type VI collagen	Interstitial matrix degradation
5	NIC	Cathepsin S degraded nidogen-1	Basement membrane degradation
6	DCN-CS	Cathepsin S degraded decorin	Proteoglycan degradation
7	VCANM	MMP degraded versican	Proteoglycan degradation
**Cross-Linking**			
8	LOX-L2	Lysyl oxidase homolog 2	Crosslinking enzyme
**Inflammation**			
9	VICM	Citrullinated and MMP degraded vimentin	Macrophage activity / inflammation
10	CRPM	MMP degraded C-reactive protein	Chronic inflammation

Legend: This table depicts the collagen biomarkers and their specifications.

Supplementary Figure 1: Baseline Biomarkers or ECM degradation (C4M, DCN-CS, NIC and VCANM) and ECM inflammation (VICM and CRPM) in the exploratory cohort

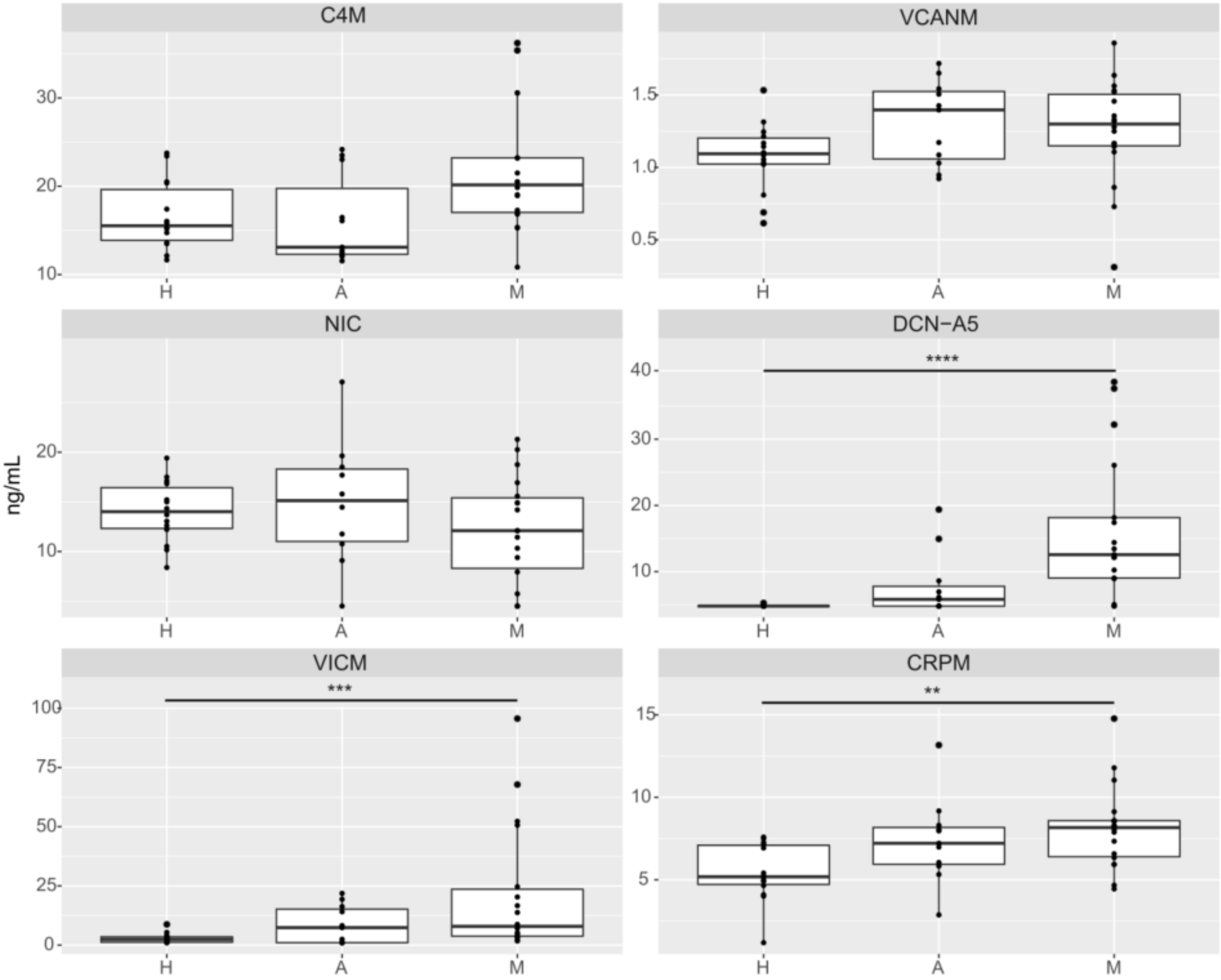

Legend: Tukey boxplots (lines representing 25th, 50th, and 75th percentile with whiskers representing lowest and highest values and *representing outliers defined as 1.5x the 75th or 25th percentile respectively) depict levels of C4M, DCN-CS, NIC, VCANM, VICM, and CRPM (median and interquartile range) in those with metastatic (M) and adjuvant (A) disease compared with healthy controls (H). P-values: *<0.05, **<0.01, ***<0.001, ****<0.0001

Supplementary Figure 2: Spearman correlation of LOXL2 with C1M, PRO-C3 and C6M

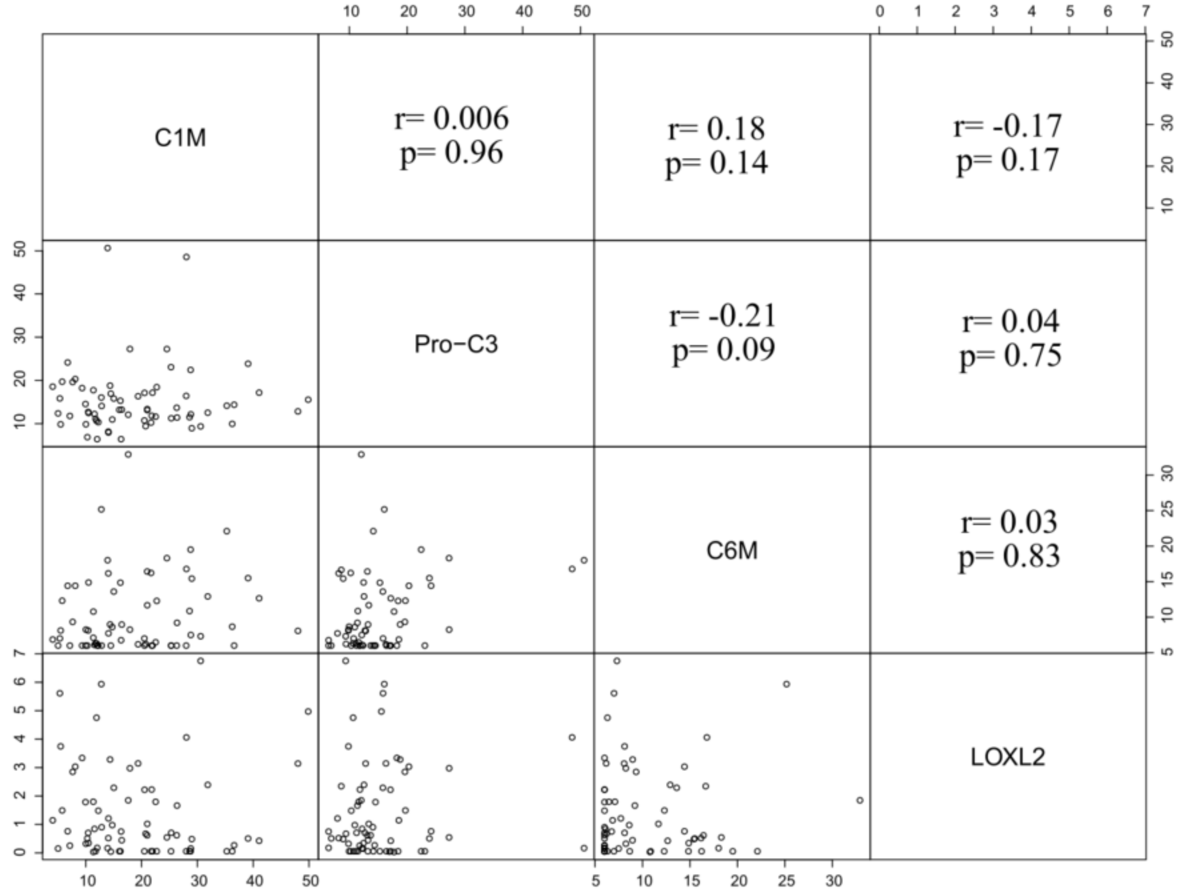

Legend: Figure depicts relationships of baseline collagen biomarkers with associated Spearman correlation coefficients and p-values.

Supplementary Figure 3: Baseline levels of C1M and Pro-C3 in the TM study by Outcome and Stage

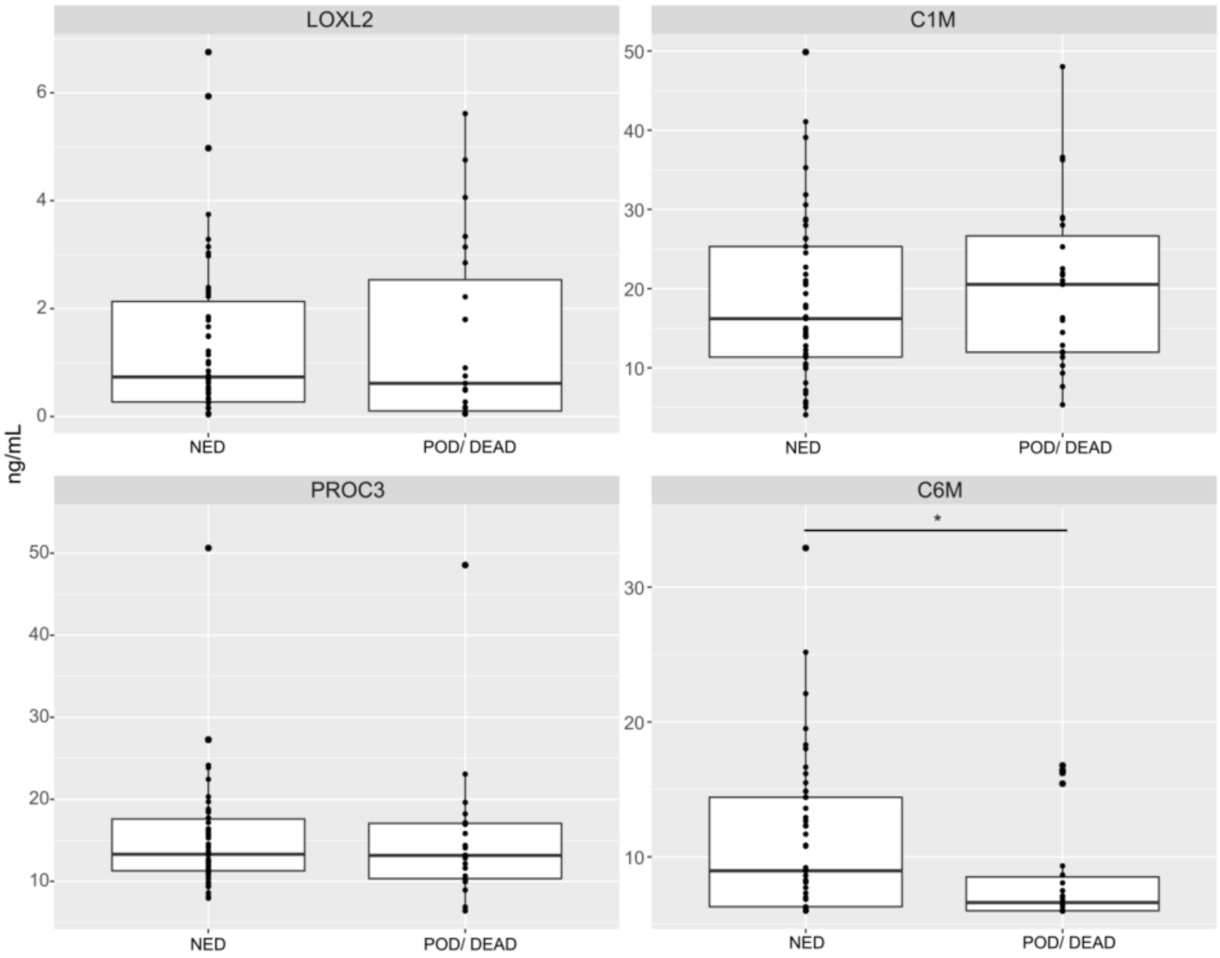

Legend: Tukey boxplots (lines representing 25th, 50th, and 75th percentile with whiskers representing lowest and highest values and *representing outliers defined as 1.5x the 75th or 25th percentile respectively) of baseline C1M and Pro-C3 levels are depicted, stratified by those who experienced POD/death vs. those who were NED. P-values: *<0.05, **<0.01, ***<0.001, ****<0.0001

Supplementary Figure 4: Cytokine Analysis in Orthotopic Mouse Models of TNBC treated with TM

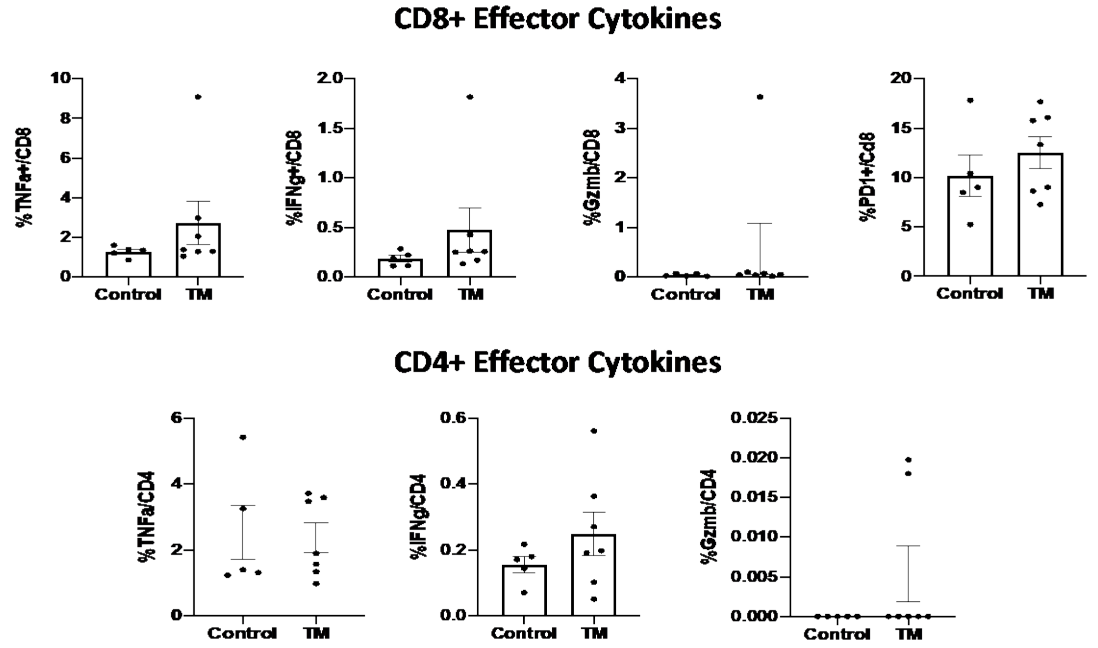

Legend. There was a trend towards higher IFN-g secretion from T cells in mice treated with TM compared with controls and a slightly higher PD-1 expression on CD8+ T cells in TM-treated mice versus controls. All error bars represent standard error of mean (SEM), and all experiemnts were run in duplicate.

Supplementary Figure 5: Gating Strategies for Flow Cytometry Analyses

A. Representative Gating Strategy for Immune Cells

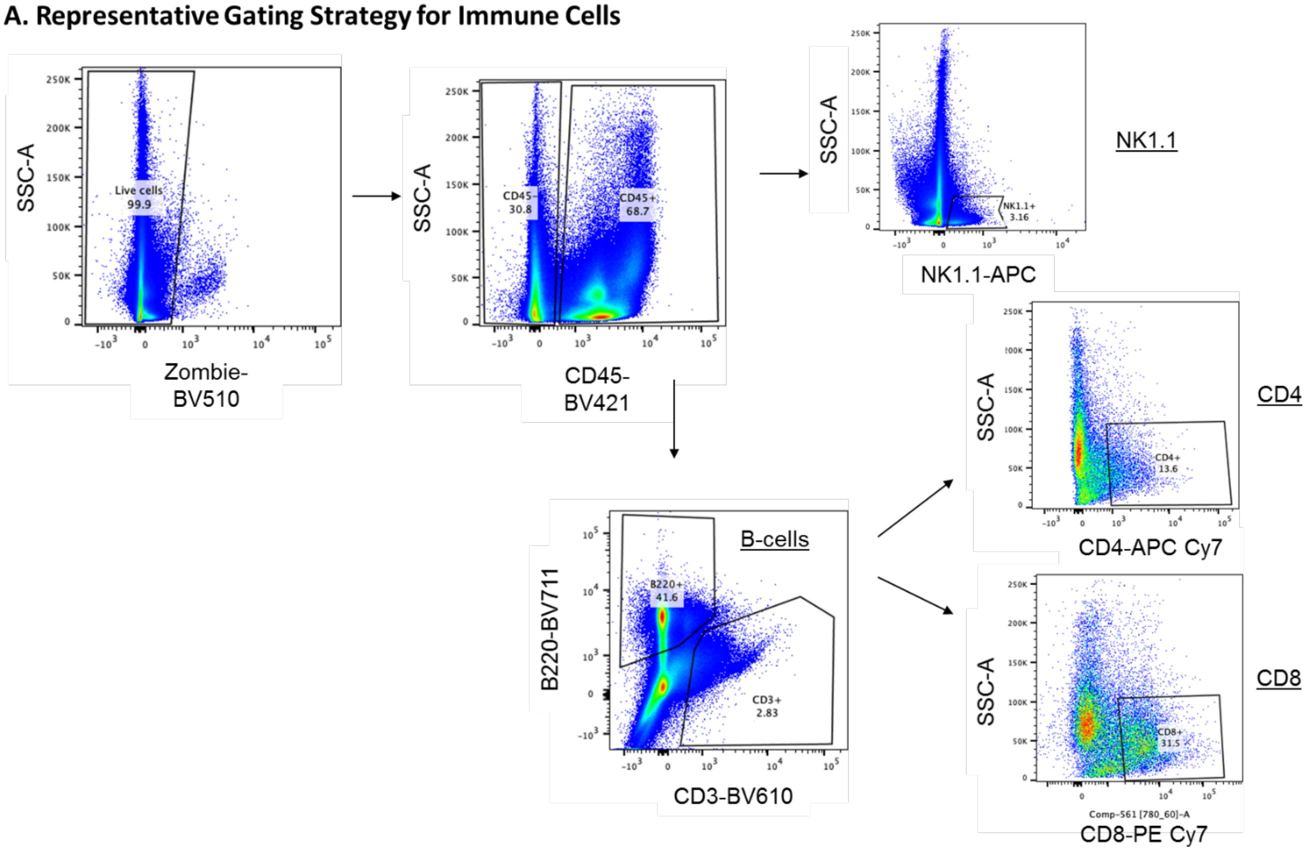

B. Representative Gating Strategy for Cytokines and PD-1

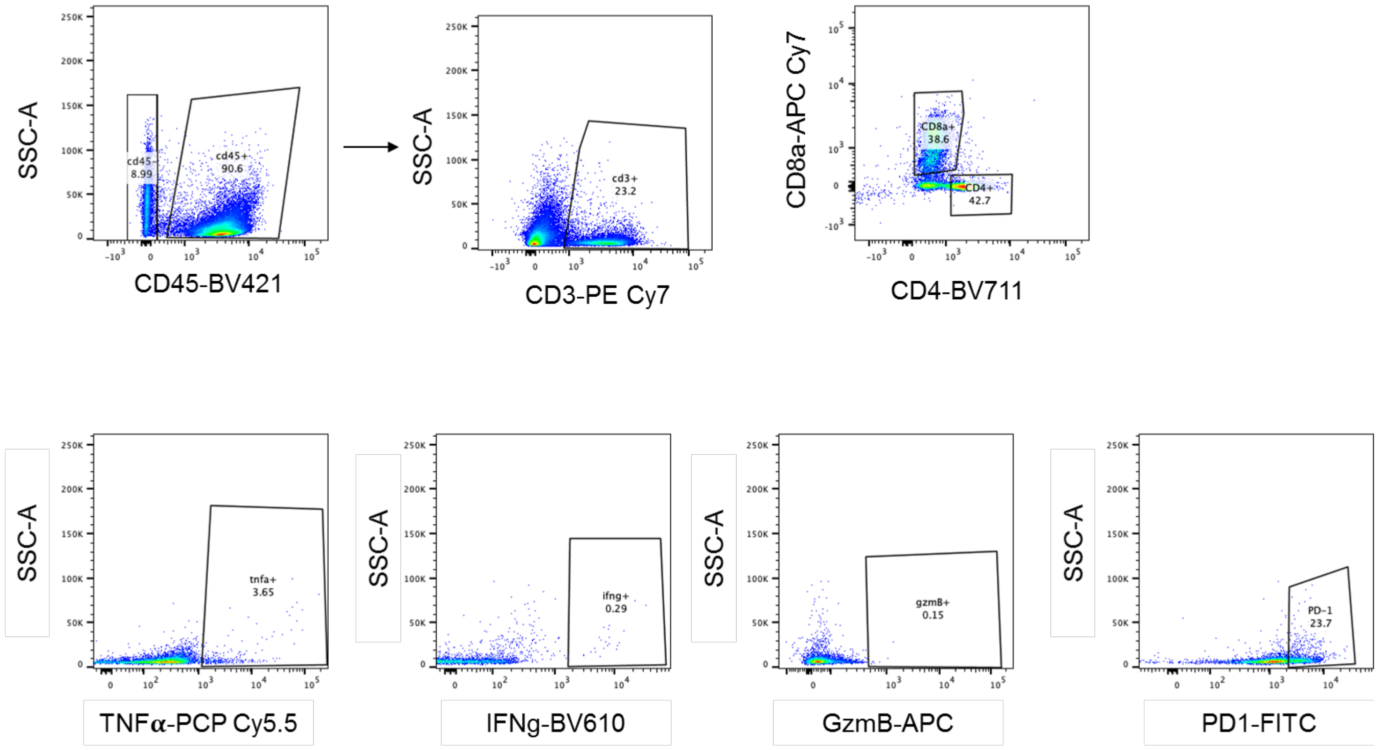

C. Representative Gating Strategy for Myeloid Immune Cells

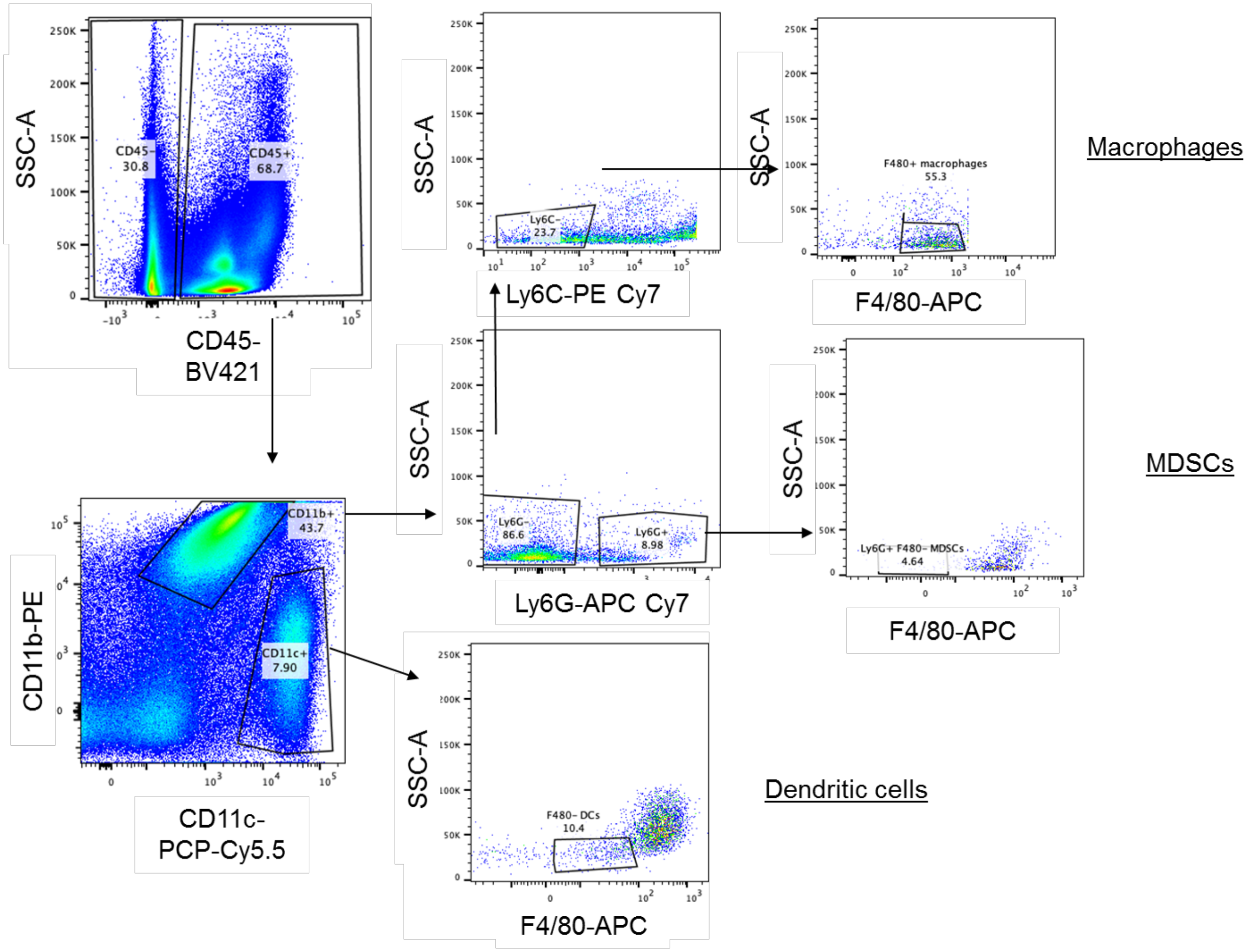

Legend: This figure depicts gating strategies for the various flow cytometry analyses. After gating the cells on SSC-A vs. FSC-A, single cells were selected on two separate parameters (FSC-H vs. FSC-W, followed by SSC-H vs. SSC-W), not shown here. The next step was to gate live cells using Zombie fixable dye, followed by CD45+, B220+, CD3+, CD4+ and CD8+ as shown (Panel A). Tumor cells were CD45-mCherry+. Gating for cytokines and PD-1 was performed for both CD4+ and CD8+ cells (Panel B). Representative gating strategy performed for myeloid cells (Panel C).
