# Supplementary material for: Tetrathiomolybdate (TM)-associated copper depletion influences collagen remodeling and immune response in the pre-metastatic niche of breast cancer
Source: NPJ Breast Cancer. 2021 Aug 23;7:108. doi: 10.1038/s41523-021-00313-w (PMC8382701; doi:10.1038/s41523-021-00313-w)
Supplement: Supplementary file 2 — Reporting Summary [file 41523_2021_313_MOESM2_ESM.pdf]

nature research

Corresponding author(s): Linda Vahdat

Last updated by author(s): 2/6/2021

Reporting Summary

Nature Research wishes to improve the reproducibility of the work that we publish. This form provides structure for consistency and transparency in reporting. For further information on Nature Research policies, see our Editorial Policies and the Editorial Policy Checklist.

Statistics

For all statistical analyses, confirm that the following items are present in the figure legend, table legend, main text, or Methods section.

n/a	Confirmed	
☐	☒	The exact sample size (n) for each experimental group/condition, given as a discrete number and unit of measurement
☒	☐	A statement on whether measurements were taken from distinct samples or whether the same sample was measured repeatedly
☐	☒	The statistical test(s) used AND whether they are one- or two-sided<br>*Only common tests should be described solely by name; describe more complex techniques in the Methods section.*
☐	☒	A description of all covariates tested
☐	☒	A description of any assumptions or corrections, such as tests of normality and adjustment for multiple comparisons
☐	☒	A full description of the statistical parameters including central tendency (e.g. means) or other basic estimates (e.g. regression coefficient) AND variation (e.g. standard deviation) or associated estimates of uncertainty (e.g. confidence intervals)
☐	☒	For null hypothesis testing, the test statistic (e.g. F, t, r) with confidence intervals, effect sizes, degrees of freedom and P value noted<br>*Give P values as exact values whenever suitable.*
☒	☐	For Bayesian analysis, information on the choice of priors and Markov chain Monte Carlo settings
☒	☐	For hierarchical and complex designs, identification of the appropriate level for tests and full reporting of outcomes
☐	☒	Estimates of effect sizes (e.g. Cohen's d, Pearson's r), indicating how they were calculated

Our web collection on statistics for biologists contains articles on many of the points above.

Software and code

Policy information about availability of computer code

Data collection	Data collection was performed per Weill Cornell Medical Center and Memorial Sloan Kettering Cancer Center regulations and stored on confidential and protected servers.
Data analysis	The statistical analyses were performed using GraphPad/Prism (version 7), STATA (version 16), and R software (3.4.1 version, R Development Core Team, 2017). Two-sided P-values <0.05 was chosen for statistical significance.

For manuscripts utilizing custom algorithms or software that are central to the research but not yet described in published literature, software must be made available to editors and reviewers. We strongly encourage code deposition in a community repository (e.g. GitHub). See the Nature Research guidelines for submitting code & software for further information.

Data

Policy information about availability of data

All manuscripts must include a data availability statement. This statement should provide the following information, where applicable:

- Accession codes, unique identifiers, or web links for publicly available datasets
- A list of figures that have associated raw data
- A description of any restrictions on data availability

The datasets generated during and/or analyzed during the current study and study protocol are available from the corresponding author on reasonable request.

Field-specific reporting

Please select the one below that is the best fit for your research. If you are not sure, read the appropriate sections before making your selection.

☒ Life sciences ☐ Behavioural & social sciences ☐ Ecological, evolutionary & environmental sciences

For a reference copy of the document with all sections, see nature.com/documents/nr-reporting-summary-flat.pdf

Life sciences study design

All studies must disclose on these points even when the disclosure is negative.

Sample size	75 women enrolled on a phase II study of TM.
Data exclusions	All women who received at least two doses of TM (n=74) were included in the survival analyses. Collagen biomarker data was available in n=69 women for this analysis, as the remaining women had insufficient samples for processing.
Replication	To assess utility of collagen biomarkers, we first tested 10 biomarkers in a discovery cohort of 91 women with breast cancer and then validated 4 of these biomarkers (LOXL2, C1M, PRO-C3 and C6M) in the TM patient cohort. Preclinical studies were conducted in 5 mice per cohort (TM and controls).
Randomization	Non-randomized study.
Blinding	No blinding

Reporting for specific materials, systems and methods

We require information from authors about some types of materials, experimental systems and methods used in many studies. Here, indicate whether each material, system or method listed is relevant to your study. If you are not sure if a list item applies to your research, read the appropriate section before selecting a response.

Materials & experimental systems

n/a	Involved in the study	
☒	☐	Antibodies
☒	☐	Eukaryotic cell lines
☒	☐	Palaeontology and archaeology
☐	☒	Animals and other organisms
☐	☒	Human research participants
☐	☒	Clinical data
☒	☐	Dual use research of concern

Methods

n/a	Involved in the study	
☒	☐	ChIP-seq
☐	☒	Flow cytometry
☒	☐	MRI-based neuroimaging

Animals and other organisms

Policy information about studies involving animals; ARRIVE guidelines recommended for reporting animal research

Laboratory animals	Mouse syngeneic TNBC cell lines EO771 (obtained from Robin Anderson lab, La Trobe University, Australia) and its lung metastatic derivative EO771.ML1 (derived according to standard protocol at Mittal lab, WCM)
Wild animals	None
Field-collected samples	None
Ethics oversight	All animal work was conducted in accordance with a protocol approved by the Institutional Animal Care and Use Committee at Weill Cornell Medicine.

Note that full information on the approval of the study protocol must also be provided in the manuscript.

Human research participants

Policy information about studies involving human research participants

Population characteristics	Patients were considered eligible for the study if they met the following criteria: at least 18 years of age; histologically confirmed stage II TNBC, stage III, or stage IV with no evidence of disease (NED) of all molecular subtypes; no radiographic, biochemical, or physical evidence of active breast cancer; more than six weeks from previous therapy including surgery,

	radiation, chemotherapy, biologic treatment; Eastern Cooperative Oncology Group (ECOG) performance status 0–1 and adequate organ function.
Recruitment	For this study, all patients who received at least 2 doses of TM (n=74) were included in the survival analysis. All patients with available samples for biomarker analysis (n=69) were included in the collagen biomarker analysis.
Ethics oversight	The study was conducted under Weill Cornell Medicine protocol #0309006307 and #0611008853, and under Memorial Sloan Kettering Cancer Center protocol #18-023, and appropriate informed consent was obtained from patients.

Note that full information on the approval of the study protocol must also be provided in the manuscript.

Clinical data

Policy information about clinical studies

All manuscripts should comply with the ICMJE guidelines for publication of clinical research and a completed CONSORT checklist must be included with all submissions.

Clinical trial registration	NCT00195091
Study protocol	Full protocol is available from the corresponding author upon request.
Data collection	Patients were accrued between June 2007 and August 2014.
Outcomes	Progression-free survival using RECIST criteria, overall survival, and adverse event reporting using CTCAE version 3.0.

Flow Cytometry

Plots

Confirm that:

☒ The axis labels state the marker and fluorochrome used (e.g. CD4-FITC).

☒ The axis scales are clearly visible. Include numbers along axes only for bottom left plot of group (a 'group' is an analysis of identical markers).

☒ All plots are contour plots with outliers or pseudocolor plots.

☒ A numerical value for number of cells or percentage (with statistics) is provided.

Methodology

Sample preparation	Described in the methods
Instrument	BD Fortessa
Software	FlowJo v10
Cell population abundance	Described in methods
Gating strategy	Supplementary data added

☒ Tick this box to confirm that a figure exemplifying the gating strategy is provided in the Supplementary Information.
